# 4′-[2-(Trifluoro­meth­yl)phen­yl]-2,2′:6′,2′′-terpyridine

**DOI:** 10.1107/S1600536809002384

**Published:** 2009-01-23

**Authors:** Peter Ledwaba, Orde Q. Munro, Kirsty Stewart

**Affiliations:** a School of Chemical and Physical Sciences, University of KwaZulu–Natal, Scottsville 3209, South Africa

## Abstract

The title compound, C_22_H_14_F_3_N_3_, is a versatile tridentate *N*-donor ligand consisting of a terpyridyl (terpy) molecule substituted in the 4′-position by a phenyl group, itself substituted in an *ortho*-position by a bulky trifluoro­methyl group. The phenyl ring is twisted as a result of steric inter­actions involving the bulky trifluoro­methyl substituent. This is reflected in the dihedral angle between the mean plane through the C atoms of the phenyl ring and the terpyridyl unit being 69.2 (1)°. The crystal structure contains no short van der Waals contacts. However, the terpy units stack in a head-to-tail orientation perpendicular to the *c* axis. The structure is is loosely stabilized by π–π inter­actions between the terminal pyridine rings of adjacent mol­ecules along the stack. The perpendicular distance between the mean planes through the terpy moieties of adjacent mol­ecules is 3.4 (1) Å.

## Related literature

For related structures, see: Bessel *et al.* (1992 ▶); Brandt *et al.* (1954 ▶); Dwyer & Mellor (1964 ▶); Field *et al.* (2002 ▶); Gillard (1983 ▶); Lindoy & Livingstone (1967 ▶); Morgan & Burstall (1932 ▶, 1934 ▶, 1938 ▶); Serpone *et al.* (1983 ▶); Storrier *et al.* (1997 ▶). For background, see  Constable *et al.* (1990 ▶, 1992 ▶); Hunter & Sanders (1990 ▶); Kröhnke (1976 ▶); Thummel & Jahng (1985 ▶).
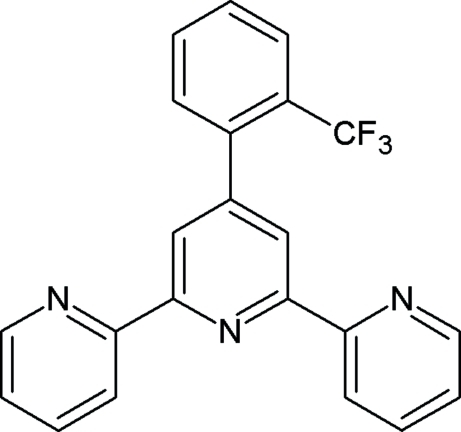


## Experimental

### 

#### Crystal data

C_22_H_14_F_3_N_3_
*M* *_r_* = 377.36
Triclinic, 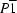

*a* = 7.767 (5) Å
*b* = 10.923 (3) Å
*c* = 11.748 (3) Å
α = 75.64 (2)°
β = 74.03 (4)°
γ = 72.93 (4)°
*V* = 900.8 (7) Å^3^
*Z* = 2
Mo *K*α radiation
μ = 0.11 mm^−1^
*T* = 293 (2) K
0.60 × 0.30 × 0.30 mm

#### Data collection

Oxford Diffraction Xcalibur2 CCD diffractometer
Absorption correction: multi-scan (*CrysAlis RED*; Oxford Diffraction, 2003 ▶) *T*_min_ = 0.930, *T*_max_ = 0.969
3953 measured reflections
3155 independent reflections
2840 reflections with *I* > 2σ(*I*)
*R*_int_ = 0.032

#### Refinement

*R*[*F* ^2^ > 2σ(*F* ^2^)] = 0.052
*wR*(*F* ^2^) = 0.148
*S* = 1.06
3155 reflections
254 parameters
H-atom parameters constrained
Δρ_max_ = 0.43 e Å^−3^
Δρ_min_ = −0.32 e Å^−3^

### 

Data collection: *CrysAlis CCD* (Oxford Diffraction, 2003 ▶); cell refinement: *CrysAlis RED* (Oxford Diffraction, 2003 ▶); data reduction: *CrysAlis RED*; program(s) used to solve structure: *SHELXS97* (Sheldrick, 2008 ▶); program(s) used to refine structure: *SHELXL97* (Sheldrick, 2008 ▶); molecular graphics: *ORTEP-3* (Farrugia, 1997 ▶); software used to prepare material for publication: *WinGX* (Farrugia, 1999 ▶).

## Supplementary Material

Crystal structure: contains datablocks I, global. DOI: 10.1107/S1600536809002384/hg2469sup1.cif

Structure factors: contains datablocks I. DOI: 10.1107/S1600536809002384/hg2469Isup2.hkl

Additional supplementary materials:  crystallographic information; 3D view; checkCIF report

## Figures and Tables

**Fig. 1.**
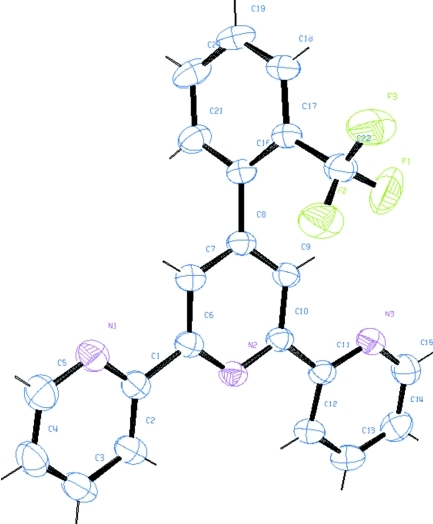
Molecular structure of 4'-(2'''-trifluoromethylphenyl)-2,2':6',2''-terpyridine, showing 50% probability displacement ellipsoids and atomic numbering.

**Fig. 2.**
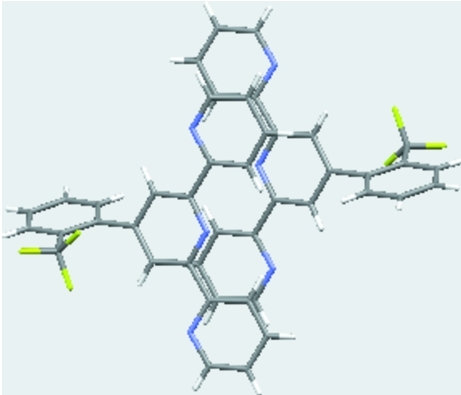
A view perpendicular to the mean plane through the atoms comprising the terpyridyl (terpy) moiety of two adjacent terpy units in the crystals of the 4'-(2'''-trifluoromethylphenyl)-2, 2':6', 2''-terpyridine ligand. Note that the successive molecules are related by a centre of inversion.

## supplementary crystallographic information

### Comment

The tridentate coordinating ligand 2,2':6',2''-terpyridine (terpy) was first isolated by Morgan & Burstall (1932, 1934, 1938) as one of the numerous products from the reaction of pyridine with iron(III) chloride.

Since the 1930s, numerous groups have examined terpy, prompted by the use of related ligands 2,2'-bipyridine (bipy) and 1,10-phenanthroline (phen), in photochemical and photophysical processes (Brandt *et al.*, 1954; Dwyer & Mellor, 1964; Gillard, 1983; Lindoy & Livingstone, 1967; Serpone *et al.*, 1983).

Reported here is the crystal structure of the tridentate terpyridyl ligand substituted in the 4'-position by a phenyl group, itself substituted in an *ortho*-position by a bulky trifluoromethyl group. *Ortho*-substitution of the 4'-phenyl ring was chosen since steric interactions between the bulky group and the 3'(5')-proton on the central pyridine ring are expected to force the 4'-substituent to rotate around the interannular bond *i.e.* the ligand will become non-planar.

In the crystal structure of 4'-(2'''-trifluoromethylphenyl)-2, 2':6',2''-terpyridine, the three pyridyl rings of the terpyridyl moiety are essentially co-planar as is preferred for maximum conjugative interaction (Thummel & Jahng, 1985). This is reflected by torsion angles between the two outer rings and the central ring of -6.5 (2)° and 9.9 (2)° for N1—C1—C6—C7 and C9—C10—C11—N3 respectively.

The terminal pyridine rings adopt a *trans*–*trans* conformation about the interannular bonds C1—C6 and C10—C11. Several derivatized terpy ligands have been found to adopt this *trans*–*trans* geometry by X-ray crystal analysis (Constable *et al.*, 1990) which is more energetically favourable when compared to other conformations as a result of the minimal nitrogen lone pair repulsions (Thummel & Jahng, 1985).

The interannular bond distances C1—C6 and C10—C11 are 1.493 (2) Å and 1.484 (2) Å respectively; these distances are comparable with the averaged values of 1.49 (1) Å and 1.49 (1) Å measured for the terpy (Bessel *et al.*, 1992) and 4'-(Ph)-terpy (Constable *et al.*, 1990) ligands respectively.

As previously postulated, the *o*-tolyl moiety is twisted about the interannular bond C8—C16, as reflected in a dihedral angle between the mean plane through the carbon atoms of the 4'-substituted and the terpyridyl moiety of 69.2 (1)°. This angle may be compared with those adopted by terpyridyl ligands containing similar substituents in the 4'-position of the terpy moiety in molecules such as the free 4'-phenyl-terpyridine (10.9°) (Constable *et al.*,  1990), 6,6''-dibromo-4'-phenyl-terpyridine (35.1°) (Constable *et al*, 1992) and 4'-(4-anilino)-terpyridine (27.2°) (Storrier *et al.*, 1997). The larger angle witnessed in the title compound is consistent with the bulky nature of the trifluoro group and the fact that it substitutes the *ortho*-position of the phenyl moiety. Clearly, substitution of a trifluoro group in the *ortho*-position of the 4'-phenyl group causes a larger rotation about the interannular bond because of steric interactions between the CF_3_ group and a hydrogen atom of the central pyridine ring that is also *ortho* with respect to the interannular bond.

There are no short van der Waals contacts less than the sum of the van der Waals radii in this system. However it is worth noting, that the terpy units stack in a head to tail orientation perpendicular to the [*c*]-axis, presumably as a result of minimizing steric interactions between the bulky trifluoromethyl substituents on adjacent molecules. However it is clear that this arrangement is not entirely successful and that poor packing does result from the presence of these bulky substituents reflected in the large solvent accessible void of 31 Å^3^. This packing orientation allows for π–π interactions between the terminal pyridine rings of adjacent molecules along the stack. The perpendicular distance between the mean planes through the terpy moieties of adjacent molecules is 3.4 (1)Å which is short enough to support π–π interactions being well within the upper distance limit of 3.8 Å for π–π interactions between organic molecules (Hunter & Sanders, 1990).

The stucture of the title compound is shown in Fig. 1. Fig. 2 shows a view perpendicular to the mean plane through the atoms comprising the terpyridyl (terpy) moiety of two adjacent terpy units in the crystals of the 4'-(2'''-trifluoromethylphenyl)-2, 2':6', 2''-terpyridine ligand. Note that the successive molecules are related by a centre of inversion.

### Experimental

4'-(2'''-trifluoromethylphenyl)-2,2':6',2''-terpyridine was synthesized by the method of Kröhnke (Field *et al.*,  2002; Kröhnke, 1976).

*N*-{1-(2'-pyridyl)-1-oxo-2-ethyl}pyridinium iodide (0.68 g, 2.2 mmol) and ammonium acetate (10 g, excess) were added to a suspension of 2-*R*-{3-(2-pyridyl)-3-oxopropenyl}benzene (2.0 mmol) in absolute ethanol (8 ml) and the mixture heated at reflux for 40 min. An off-white solid precipitated on cooling. This was collected by filtration, washed with 50% aqueous ethanol and dried *in vacuo*. Recrystallization from ethanol afforded colourless crystals of the desired ligands.

Yield: (0.41 g, 54%). m.p. (148 °C). Anal. (Calcd. For C_22_H_14_F_3_N_3_: C 70.0; H 3.7; N 11.1. Found: C 69.9; H 3.9; N 11.0%). MS(EI) *m/z*: 377, *M*^+^). ^1^H NMR (CDCl_3_): [δ 8.72 (m, 2H, H_6,6''_); 8.70 (m, 2H, H_3,3''_); 8.54 (s, 2H, H_3',5'_); 7.84 (m, 2H, H_4,4''_); 7.54 (m, 4H, C_6_H_4_); 7.35 (m, 2 H, H_5,5''_)]. UV/vis (CH_3_CN): λ_max_/nm (ε/*M*^-1 ^cm^-1^): [303 (sh, 1.3 × 10^4^); 277 (2.9 × 10^4^); 239 (3.4 × 10^4^); 208 (3.6 × 10^4^)].

### Refinement

All H atoms were positioned in geometrically idealized positions and constrained to ride on their parent atoms with C—H distances in the range 0.95–1.00 Å. and *U*_iso_(H) = 1.2–1.5*U*_eq_(C).

### Crystal data

**Table:**

C_22_H_14_F_3_N_3_	*Z* = 2
*M**_r_* = 377.36	*F*(000) = 388
Triclinic, *P*1	*D*_x_ = 1.391 Mg m^−^^3^
Hall symbol: -P 1	Melting point: 421.15 K
*a* = 7.767 (5) Å	Mo *K*α radiation, λ = 0.71073 Å
*b* = 10.923 (3) Å	Cell parameters from 3155 reflections
*c* = 11.748 (3) Å	θ = 2.4–25°
α = 75.64 (2)°	µ = 0.11 mm^−^^1^
β = 74.03 (4)°	*T* = 293 K
γ = 72.93 (4)°	Square planar, colourless
*V* = 900.8 (7) Å^3^	0.60 × 0.30 × 0.30 mm

### Data collection

**Table:**

Oxford Diffraction Xcalibur2 CCD diffractometer	3155 independent reflections
Radiation source: Enhance (Mo)X-Ray Source	2840 reflections with *I* > 2σ(*I*)
graphite	*R*_int_ = 0.032
Detector resolution: 8.4190 pixels mm^-1^	θ_max_ = 25.0°, θ_min_ = 2.4°
ω/2θ scans	*h* = −4→9
Absorption correction: multi-scan (*CrysAlis RED*; Oxford Diffraction, 2003)	*k* = −12→12
*T*_min_ = 0.930, *T*_max_ = 0.969	*l* = −13→13
3953 measured reflections	

### Refinement

**Table:**

Refinement on *F*^2^	Primary atom site location: structure-invariant direct methods
Least-squares matrix: full	Secondary atom site location: difference Fourier map
*R*[*F*^2^ > 2σ(*F*^2^)] = 0.052	Hydrogen site location: inferred from neighbouring sites
*wR*(*F*^2^) = 0.148	H-atom parameters constrained
*S* = 1.06	*w* = 1/[σ^2^(*F*_o_^2^) + (0.0902*P*)^2^ + 0.255*P*] where *P* = (*F*_o_^2^ + 2*F*_c_^2^)/3
3155 reflections	(Δ/σ)_max_ < 0.001
254 parameters	Δρ_max_ = 0.43 e Å^−^^3^
0 restraints	Δρ_min_ = −0.31 e Å^−^^3^

### Special details

**Table:**

Experimental. CrysAlis RED, Oxford Diffraction Ltd., Version 170. Empirical absorption correction using spherical harmonics, implemented in SCALE3 ABSPACK scaling algorithm.
Geometry. All e.s.d.'s (except the e.s.d. in the dihedral angle between two l.s. planes) are estimated using the full covariance matrix. The cell e.s.d.'s are taken into account individually in the estimation of e.s.d.'s in distances, angles and torsion angles; correlations between e.s.d.'s in cell parameters are only used when they are defined by crystal symmetry. An approximate (isotropic) treatment of cell e.s.d.'s is used for estimating e.s.d.'s involving l.s. planes.
Refinement. Refinement of *F*^2^ against ALL reflections. The weighted *R*-factor *wR* and goodness of fit *S* are based on *F*^2^, conventional *R*-factors *R* are based on *F*, with *F* set to zero for negative *F*^2^. The threshold expression of *F*^2^ > σ(*F*^2^) is used only for calculating *R*-factors(gt) *etc*. and is not relevant to the choice of reflections for refinement. *R*-factors based on *F*^2^ are statistically about twice as large as those based on *F*, and *R*- factors based on ALL data will be even larger.

### Fractional atomic coordinates and isotropic or equivalent isotropic displacement parameters (Å^2^)

**Table:**

	*x*	*y*	*z*	*U*_iso_*/*U*_eq_	
F1	0.5317 (2)	0.91636 (13)	0.35582 (14)	0.0845 (5)	
F2	0.71339 (15)	0.74840 (14)	0.42847 (12)	0.0725 (4)	
F3	0.4702 (2)	0.83636 (18)	0.54344 (13)	0.0926 (6)	
N1	1.1281 (2)	0.32563 (14)	0.24320 (14)	0.0467 (4)	
N2	1.02318 (17)	0.64584 (13)	0.06447 (11)	0.0340 (3)	
N3	0.79020 (19)	0.97634 (14)	−0.03608 (13)	0.0426 (4)	
C1	1.1511 (2)	0.42440 (15)	0.15132 (14)	0.0357 (4)	
C2	1.3124 (2)	0.41901 (18)	0.06241 (16)	0.0429 (4)	
H2	1.3249	0.4894	−0.0002	0.0564 (15)*	
C3	1.4538 (2)	0.30759 (19)	0.06833 (18)	0.0511 (5)	
H3	1.5638	0.3026	0.0106	0.0564 (15)*	
C4	1.4302 (3)	0.20463 (19)	0.1601 (2)	0.0536 (5)	
H4	1.5217	0.1275	0.1648	0.0564 (15)*	
C5	1.2666 (3)	0.21853 (19)	0.2456 (2)	0.0549 (5)	
H5	1.2518	0.1491	0.3088	0.0564 (15)*	
C6	0.9941 (2)	0.54241 (15)	0.14861 (13)	0.0333 (3)	
C7	0.8263 (2)	0.54237 (16)	0.23130 (14)	0.0363 (4)	
H7	0.8088	0.4674	0.2867	0.0564 (15)*	
C8	0.6857 (2)	0.65547 (16)	0.22996 (13)	0.0341 (4)	
C9	0.7156 (2)	0.76331 (15)	0.14389 (14)	0.0345 (4)	
H9	0.6248	0.8409	0.1413	0.0564 (15)*	
C10	0.8849 (2)	0.75402 (15)	0.06062 (13)	0.0328 (4)	
C11	0.9179 (2)	0.86326 (15)	−0.04004 (14)	0.0334 (4)	
C12	1.0712 (2)	0.84656 (17)	−0.13472 (15)	0.0410 (4)	
H12	1.1568	0.7666	−0.1354	0.0564 (15)*	
C13	1.0949 (2)	0.94940 (19)	−0.22719 (16)	0.0476 (4)	
H13	1.1972	0.9404	−0.2909	0.0564 (15)*	
C14	0.9649 (3)	1.06607 (18)	−0.22414 (17)	0.0495 (4)	
H14	0.9767	1.1375	−0.2856	0.0564 (15)*	
C15	0.8167 (3)	1.07390 (18)	−0.12736 (17)	0.0492 (4)	
H15	0.7290	1.1529	−0.1257	0.0564 (15)*	
C16	0.5028 (2)	0.65338 (15)	0.31581 (13)	0.0341 (4)	
C17	0.4308 (2)	0.72287 (15)	0.41048 (14)	0.0351 (4)	
C18	0.2588 (2)	0.71521 (17)	0.48555 (15)	0.0414 (4)	
H18	0.2120	0.7612	0.5488	0.0564 (15)*	
C19	0.1581 (2)	0.64052 (19)	0.46690 (16)	0.0473 (4)	
H19	0.0426	0.6373	0.5163	0.0564 (15)*	
C20	0.2281 (3)	0.5707 (2)	0.37529 (17)	0.0526 (5)	
H20	0.1606	0.5192	0.3631	0.0564 (15)*	
C21	0.3993 (2)	0.57662 (19)	0.30069 (16)	0.0459 (4)	
H21	0.4459	0.5282	0.2392	0.0564 (15)*	
C22	0.5349 (2)	0.80526 (19)	0.43493 (16)	0.0481 (4)	

### Atomic displacement parameters (Å^2^)

**Table:**

	*U*^11^	*U*^22^	*U*^33^	*U*^12^	*U*^13^	*U*^23^
F1	0.1039 (11)	0.0570 (8)	0.1051 (11)	−0.0432 (8)	−0.0206 (9)	−0.0113 (7)
F2	0.0405 (6)	0.1046 (10)	0.0925 (10)	−0.0248 (6)	−0.0147 (6)	−0.0448 (8)
F3	0.0829 (10)	0.1468 (14)	0.0763 (9)	−0.0633 (10)	0.0233 (7)	−0.0738 (10)
N1	0.0386 (8)	0.0444 (8)	0.0533 (9)	−0.0056 (6)	−0.0115 (6)	−0.0057 (7)
N2	0.0272 (6)	0.0400 (7)	0.0361 (7)	−0.0077 (5)	−0.0057 (5)	−0.0112 (6)
N3	0.0359 (7)	0.0409 (8)	0.0466 (8)	−0.0062 (6)	−0.0045 (6)	−0.0089 (6)
C1	0.0325 (8)	0.0396 (9)	0.0391 (8)	−0.0072 (6)	−0.0108 (6)	−0.0128 (7)
C2	0.0349 (8)	0.0485 (10)	0.0443 (9)	−0.0048 (7)	−0.0064 (7)	−0.0154 (7)
C3	0.0331 (9)	0.0588 (11)	0.0607 (11)	−0.0009 (8)	−0.0070 (8)	−0.0257 (9)
C4	0.0388 (9)	0.0459 (10)	0.0775 (13)	0.0035 (8)	−0.0220 (9)	−0.0197 (9)
C5	0.0469 (10)	0.0452 (10)	0.0688 (13)	−0.0050 (8)	−0.0202 (9)	−0.0027 (9)
C6	0.0296 (8)	0.0401 (8)	0.0333 (8)	−0.0085 (6)	−0.0074 (6)	−0.0113 (6)
C7	0.0326 (8)	0.0414 (9)	0.0351 (8)	−0.0102 (6)	−0.0063 (6)	−0.0071 (6)
C8	0.0277 (7)	0.0436 (9)	0.0335 (8)	−0.0112 (6)	−0.0034 (6)	−0.0121 (6)
C9	0.0268 (7)	0.0377 (8)	0.0393 (8)	−0.0069 (6)	−0.0043 (6)	−0.0120 (6)
C10	0.0268 (7)	0.0396 (8)	0.0353 (8)	−0.0099 (6)	−0.0053 (6)	−0.0119 (6)
C11	0.0267 (7)	0.0396 (8)	0.0372 (8)	−0.0093 (6)	−0.0081 (6)	−0.0105 (6)
C12	0.0301 (8)	0.0476 (9)	0.0420 (9)	−0.0079 (7)	−0.0043 (6)	−0.0081 (7)
C13	0.0384 (9)	0.0601 (11)	0.0399 (9)	−0.0157 (8)	−0.0014 (7)	−0.0046 (8)
C14	0.0521 (10)	0.0501 (10)	0.0454 (10)	−0.0181 (8)	−0.0133 (8)	0.0029 (8)
C15	0.0485 (10)	0.0401 (9)	0.0533 (10)	−0.0060 (8)	−0.0103 (8)	−0.0053 (8)
C16	0.0275 (7)	0.0402 (8)	0.0340 (8)	−0.0107 (6)	−0.0053 (6)	−0.0043 (6)
C17	0.0270 (7)	0.0403 (8)	0.0362 (8)	−0.0086 (6)	−0.0045 (6)	−0.0058 (6)
C18	0.0302 (8)	0.0497 (9)	0.0382 (8)	−0.0086 (7)	−0.0001 (6)	−0.0070 (7)
C19	0.0303 (8)	0.0657 (11)	0.0426 (9)	−0.0209 (8)	−0.0033 (7)	0.0019 (8)
C20	0.0468 (10)	0.0716 (13)	0.0498 (10)	−0.0368 (9)	−0.0091 (8)	−0.0042 (9)
C21	0.0462 (9)	0.0571 (11)	0.0415 (9)	−0.0253 (8)	−0.0032 (7)	−0.0132 (8)
C22	0.0403 (9)	0.0588 (11)	0.0489 (10)	−0.0186 (8)	0.0036 (7)	−0.0235 (9)

### Geometric parameters (Å, °)

**Table:**

F1—C22	1.331 (2)	C9—C10	1.400 (2)
F2—C22	1.332 (2)	C9—H9	0.9300
F3—C22	1.325 (2)	C10—C11	1.484 (2)
N1—C5	1.337 (2)	C11—C12	1.390 (2)
N1—C1	1.338 (2)	C12—C13	1.372 (2)
N2—C6	1.333 (2)	C12—H12	0.9300
N2—C10	1.344 (2)	C13—C14	1.376 (3)
N3—C15	1.330 (2)	C13—H13	0.9300
N3—C11	1.340 (2)	C14—C15	1.377 (3)
C1—C2	1.389 (2)	C14—H14	0.9300
C1—C6	1.493 (2)	C15—H15	0.9300
C2—C3	1.381 (3)	C16—C21	1.388 (2)
C2—H2	0.9300	C16—C17	1.398 (2)
C3—C4	1.368 (3)	C17—C18	1.397 (2)
C3—H3	0.9300	C17—C22	1.493 (2)
C4—C5	1.381 (3)	C18—C19	1.372 (3)
C4—H4	0.9300	C18—H18	0.9300
C5—H5	0.9300	C19—C20	1.370 (3)
C6—C7	1.395 (2)	C19—H19	0.9300
C7—C8	1.388 (2)	C20—C21	1.387 (3)
C7—H7	0.9300	C20—H20	0.9300
C8—C9	1.380 (2)	C21—H21	0.9300
C8—C16	1.501 (2)		
			
C5—N1—C1	117.21 (16)	C13—C12—C11	119.27 (16)
C6—N2—C10	118.17 (13)	C13—C12—H12	120.4
C15—N3—C11	116.89 (15)	C11—C12—H12	120.4
N1—C1—C2	122.33 (15)	C12—C13—C14	118.93 (16)
N1—C1—C6	116.64 (15)	C12—C13—H13	120.5
C2—C1—C6	121.03 (15)	C14—C13—H13	120.5
C3—C2—C1	119.02 (17)	C13—C14—C15	118.00 (16)
C3—C2—H2	120.5	C13—C14—H14	121.0
C1—C2—H2	120.5	C15—C14—H14	121.0
C4—C3—C2	119.23 (17)	N3—C15—C14	124.54 (17)
C4—C3—H3	120.4	N3—C15—H15	117.7
C2—C3—H3	120.4	C14—C15—H15	117.7
C3—C4—C5	118.07 (17)	C21—C16—C17	117.94 (14)
C3—C4—H4	121.0	C21—C16—C8	117.85 (14)
C5—C4—H4	121.0	C17—C16—C8	124.21 (14)
N1—C5—C4	124.10 (19)	C18—C17—C16	120.11 (15)
N1—C5—H5	118.0	C18—C17—C22	118.44 (15)
C4—C5—H5	118.0	C16—C17—C22	121.44 (14)
N2—C6—C7	122.53 (15)	C19—C18—C17	120.60 (16)
N2—C6—C1	116.83 (14)	C19—C18—H18	119.7
C7—C6—C1	120.63 (15)	C17—C18—H18	119.7
C8—C7—C6	119.27 (15)	C20—C19—C18	119.89 (15)
C8—C7—H7	120.4	C20—C19—H19	120.1
C6—C7—H7	120.4	C18—C19—H19	120.1
C9—C8—C7	118.43 (14)	C19—C20—C21	120.07 (16)
C9—C8—C16	122.38 (14)	C19—C20—H20	120.0
C7—C8—C16	119.04 (15)	C21—C20—H20	120.0
C8—C9—C10	118.93 (14)	C20—C21—C16	121.37 (17)
C8—C9—H9	120.5	C20—C21—H21	119.3
C10—C9—H9	120.5	C16—C21—H21	119.3
N2—C10—C9	122.57 (15)	F3—C22—F2	105.78 (17)
N2—C10—C11	116.41 (13)	F3—C22—F1	107.02 (17)
C9—C10—C11	120.99 (14)	F2—C22—F1	104.80 (16)
N3—C11—C12	122.37 (15)	F3—C22—C17	112.81 (14)
N3—C11—C10	116.52 (14)	F2—C22—C17	113.43 (15)
C12—C11—C10	121.08 (14)	F1—C22—C17	112.38 (16)
			
C5—N1—C1—C2	−1.2 (2)	C9—C10—C11—C12	−168.23 (14)
C5—N1—C1—C6	178.95 (15)	N3—C11—C12—C13	0.7 (2)
N1—C1—C2—C3	0.4 (2)	C10—C11—C12—C13	178.79 (15)
C6—C1—C2—C3	−179.75 (14)	C11—C12—C13—C14	−0.7 (3)
C1—C2—C3—C4	1.2 (3)	C12—C13—C14—C15	0.3 (3)
C2—C3—C4—C5	−2.0 (3)	C11—N3—C15—C14	0.0 (3)
C1—N1—C5—C4	0.4 (3)	C13—C14—C15—N3	0.1 (3)
C3—C4—C5—N1	1.2 (3)	C9—C8—C16—C21	109.60 (18)
C10—N2—C6—C7	0.5 (2)	C7—C8—C16—C21	−65.9 (2)
C10—N2—C6—C1	−179.33 (12)	C9—C8—C16—C17	−70.9 (2)
N1—C1—C6—N2	173.37 (13)	C7—C8—C16—C17	113.59 (18)
C2—C1—C6—N2	−6.5 (2)	C21—C16—C17—C18	−0.8 (2)
N1—C1—C6—C7	−6.5 (2)	C8—C16—C17—C18	179.76 (14)
C2—C1—C6—C7	173.69 (14)	C21—C16—C17—C22	178.55 (16)
N2—C6—C7—C8	−2.7 (2)	C8—C16—C17—C22	−0.9 (2)
C1—C6—C7—C8	177.13 (13)	C16—C17—C18—C19	−0.5 (2)
C6—C7—C8—C9	1.9 (2)	C22—C17—C18—C19	−179.83 (16)
C6—C7—C8—C16	177.55 (13)	C17—C18—C19—C20	1.3 (3)
C7—C8—C9—C10	0.9 (2)	C18—C19—C20—C21	−0.7 (3)
C16—C8—C9—C10	−174.63 (13)	C19—C20—C21—C16	−0.6 (3)
C6—N2—C10—C9	2.5 (2)	C17—C16—C21—C20	1.3 (3)
C6—N2—C10—C11	−175.44 (12)	C8—C16—C21—C20	−179.21 (17)
C8—C9—C10—N2	−3.2 (2)	C18—C17—C22—F3	15.5 (2)
C8—C9—C10—C11	174.63 (13)	C16—C17—C22—F3	−163.81 (17)
C15—N3—C11—C12	−0.4 (2)	C18—C17—C22—F2	135.79 (17)
C15—N3—C11—C10	−178.53 (14)	C16—C17—C22—F2	−43.6 (2)
N2—C10—C11—N3	−172.12 (13)	C18—C17—C22—F1	−105.57 (18)
C9—C10—C11—N3	9.9 (2)	C16—C17—C22—F1	75.1 (2)
N2—C10—C11—C12	9.7 (2)		
